# IMPLEMENTATION AND EFFECTIVENESS OF VIRTUAL REALITY PROGRAMS IN LONG-TERM CARE SETTINGS

**DOI:** 10.1093/geroni/igad104.1050

**Published:** 2023-12-21

**Authors:** Marie Savundranayagam, Kristine Williams

## Abstract

Virtual reality (VR) is an immersive technology that allows users to experience a computer-generated simulation of a three-dimensional environment. It is used in healthcare for education and training purposes, as therapeutic interventions, and for social engagement. However, there are few studies exploring the use of VR in long-term care settings. The purpose of this symposium is to share lessons learned on the implementation and effectiveness of VR programs in long-term care settings. All studies use the Consolidated Framework for Implementation Research to guide their exploration of the facilitators and barriers to implementation. They use qualitative methods, such as interviews, focus groups, and ethnographic observation, to collect data on the experiences of staff and residents participating in the programs. Study one highlights the critical need to engage decision makers to assess organizational readiness prior to creating an implementation strategy. Study two showcases the iterative process involved in translating an in-person, person-centered communication training into a VR training program for frontline healthcare workers. Study three features the benefits and challenges of using VR with older residents. Study four explores staff perspectives on facilitators and barriers to adopting VR in long-term care. The studies highlight the potential benefits of VR in enhancing communication skills, social engagement, and support for residents’ personhood. They also identify factors for successful implementation, such as staff champions, sufficient technological infrastructure, and organizational support for infrastructure and human resources. The studies offer valuable insights for researchers and practitioners working to implement novel VR interventions in long-term care settings.

